# On the Evolution of Nano-Structures at the Al–Cu Interface and the Influence of Annealing Temperature on the Interfacial Strength

**DOI:** 10.3390/nano12203658

**Published:** 2022-10-18

**Authors:** Xiaoli Wang, Guang Cheng, Yang Zhang, Yuxin Wang, Wenjun Liao, T. A. Venkatesh

**Affiliations:** 1 College of Mechanical and Electrical Engineering, Beijing University of Chemical Technology, Beijing 100029, China; 2 School of Materials Science and Engineering, Jiangsu University of Science and Technology, Zhenjiang 212003, China; 3 Central Research Academy, Shanghai Electric Group Co., Ltd., Shanghai 200070, China; 4 Department of Materials Science and Chemical Engineering, Stony Brook University, Stony Brook, NY 11794, USA

**Keywords:** indentation, interfacial strength, Al–Cu interface, molecular dynamics

## Abstract

Molecular dynamics (MD) simulations are invoked to simulate the diffusion process and microstructural evolution at the solid–liquid, cast-rolled Al–Cu interfaces. K-Means clustering algorithm is used to identify the formation and composition of two types of nanostructural features in the Al-rich and Cu-rich regions of the interface (i.e., the intermetallic Al_2_Cu near the Al-rich interface and the intermetallic Al_4_Cu_9_ near the Cu-rich interface). MD simulations are also used to assess the effects of annealing temperature on the evolution of the compositionally graded microstructural features at the Al–Cu interfaces and to characterize the mechanical strength of the Al–Cu interfaces. It is found that the failure of the Al–Cu interface takes place at the Al-rich side of the interface (Al_2_Cu–Al) which is mechanically weaker than the Cu-rich side of the interface (Cu–Al_4_Cu_9_), which is also verified by the nanoindentation studies of the interfaces. Centrosymmetry parameter analyses and dislocation analyses are used to understand the microstructural features that influence deformation behavior leading to the failure of the Al–Cu interfaces. Increasing the annealing temperature reduces the stacking fault density at the Al–Cu interface, suppresses the generation of nanovoids which are precursors for the initiation of fracture at the Al-rich interface, and increases the strength of the interface.

## 1. Introduction

High interfacial strength is desired in various techniques for joining and bonding materials. In recent years, with significant demands for light-weighting as a means to reduce energy costs in vehicles and electrical equipment, advanced joining and welding technologies (e.g., friction stir welding), have been developed and implemented, enabling the joining of lightweight materials (such as aluminum) with other materials with a range of thermophysical properties. Depending on the materials involved in the joining process, the evolution of the microstructures near the interface could be quite complex. For example, the interface generated in the joining process for aluminum or copper generally contains a graded microstructure with a certain amount of intermetallic phases, such as Al_7_Fe_2_Si [1], Cu_6_Sn_5_ and Cu_3_Sn [2], and Al_4_Cu_9_ and Al_2_Cu [3], near the interfaces which are much stiffer and stronger than the base material. However, the bonding strength of the interfaces between the pure metal and these intermetallic compounds is often lower than the strength of the base materials or compounds. Therefore, there is a recognition that by enhancing the quality of the interface, reducing the defects near the interface, and by controlling the size and morphologies of the intermetallic phases in the interface regions, the bonding strength and durability of the bonded structure can be enhanced [4].

The interface regions that are generated in the joining process are generally very small, around 5–40 μm [5,6,7]. Furthermore, the interfaces could have a wavy morphology which increases the difficulty in directly and accurately characterizing the local strength of the interfaces. Within this context, due to their versatility and non-destructive nature, instrumented indentation-based tests have been extensively implemented to investigate the interface obtained in joining dissimilar materials (e.g., Al–Mg, Al–Fe, Sn–Cu, or Sn–Cu–Ag systems). Indentation experiments have provided insights into the local variation of mechanical properties near the interface regions [8,9,10,11,12] which could be as fine as about 100 nm. However, very few studies have focused on providing insights into the strength of the interfaces. It is necessary to consider the intermetallic compounds that are generated in the joining and the processing as well.

On the modeling side, in recent years, the molecular dynamics (MD) method has been adapted to study the strength and deformation mechanisms of various nanostructures [13,14,15,16,17,18]. Within the context of two-phase or multi-phase material systems, the atomic structure-based approach has been successfully invoked to study the evolution of interface characteristics, such as interface thickness and strength, and correlate the interface characteristics with the processing parameters, such as pressure and temperature that are used in the processing of such materials. Accurate mesoscale models have been implemented in analyzing the mechanical performance of CuSn [2] and fine-grained high-strength steels [19]. With the newly developed interaction potential for MD methods, the strengths of binary and ternary systems, such as Al–Cu [20,21], Cu–Zr [22], Ti–V–N [23], Cr–Fe [24], and high entropy alloy systems [25,26] have been studied as well. However, there are very few comprehensive studies that provide insights into the failure processes that are initiated at the interfaces.

Hence, the objectives of the present study are:

(i) To develop a modeling framework that captures the nano-scale diffusion process that occurs during the joining of a model Al–Cu system;
(ii) To predict the evolution of microstructural features such as defects at the interfaces and intermetallic compound formation near the interfaces;
(iii) To assess the effects of annealing on the quality of the interfaces;
(iv) To characterize the tensile and shear strength of the interfaces, and to understand the failure mechanisms that occur at the interfaces;
(v) To correlate modeling predictions for the strength of interfaces with indentation experiments.

## 2. Interface Preparation and Characterization

**Figure 1:**
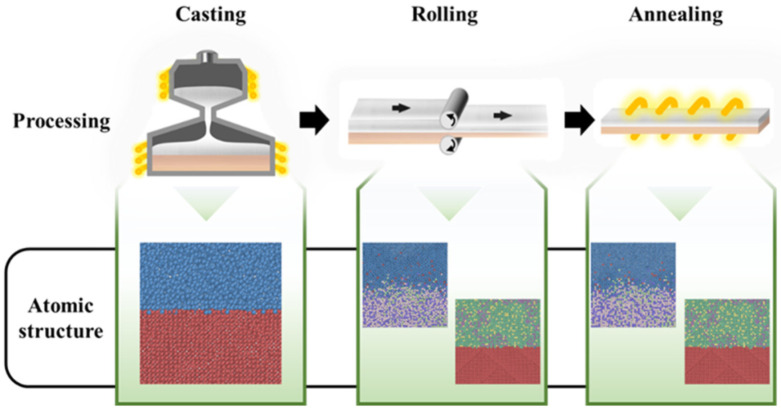
The sketch of Al–Cu cast rolling processing (including casting, cooling and rolling, and annealing) and corresponding atomic structures (including Al, Cu, and intermetallic compounds).

The model Al–Cu bi-metallic plate was prepared from 99% industry pure aluminum and 99% pure oxygen-free copper. The plate was fabricated by the solid–liquid cast-rolling method, as illustrated in Figure 1. Before the sample preparation, the copper surface was cleaned. The sample preparation included two steps. First, the cleaned copper tube was heated to 420 °C. The aluminum was melted at 710 °C, cast into the copper tube, and cooled to form the Al–Cu bimetal. Second, the heated Al–Cu composite plate was rolled to a 60 × 30 × 300 mm composite plate, followed by an annealing process. The preheating temperature, casting temperature, rolling reduction, and annealing temperature are factors that could influence the Al–Cu interface thickness, properties, and performance [27,28].

The phase structures of Al–Cu interfaces near the Al side and the Cu side were detected by X-ray diffraction (Bruker D8 advance, Karlsruhe, Germany). The evolution of microstructures and the primary element analyses near the interface were conducted using a scanning electron microscope (SEM, JEOL JSM-5510, Tokoy, Japan). The element analyses were performed using an electron probe microanalyzer (EPMA, JEOL JXA-8530F, Tokoy, Japan) at two locations. The variation in mechanical properties near the interface was characterized by microindentation hardness testing using a microindenter (QNESS Q10A, Salzburg, Austria) at 25 g load and 15 s holding time. Furthermore, nanoindentation testing near the interface was conducted using an instrumental indentation machine (Hysitron TI950 Triboindenter, Minneapolis, MN, United States) with a diamond Berkovich indenter, and a maximum indentation load of 6000 μN.

## 3. Experimental Results

**Figure 2:**
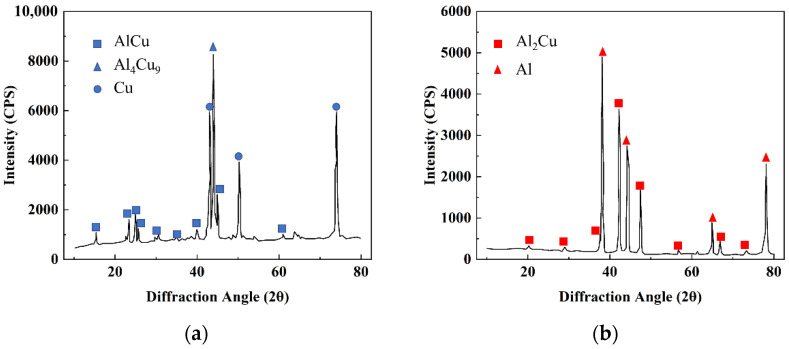
The X-ray diffraction patterns and the identified phases of the Al–Cu interface at two different locations: (**a**) Cu side and (**b**) Al side.

**Figure 3:**
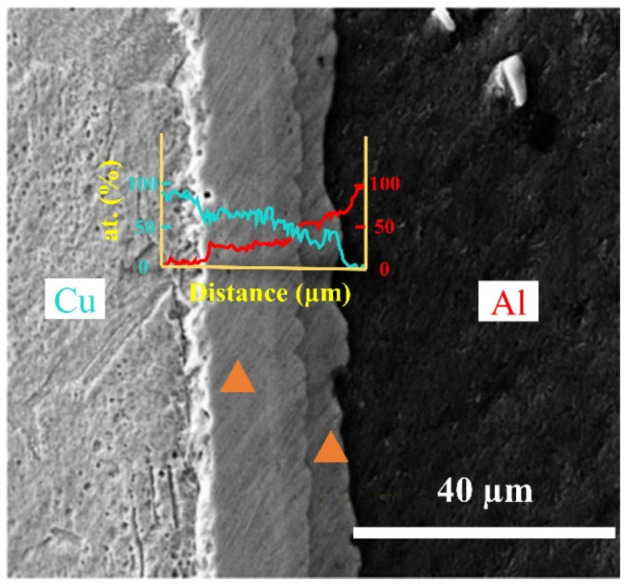
The SEM images of the Al–Cu interface (blue and red lines denote the at.% of Cu and Al, respectively) with locations of the EPMA (highlighted in the triangles).

The locations at which diffraction images were taken and the electron probe microanalysis was conducted are shown in Figure 2. The results of the compositional analysis are presented in Table 1. From the compositional analysis, it is clear that the interface near the Cu side was dominated by Al_4_Cu_9_ intermetallic and a small amount of Cu. Small amounts of C and O were also detected, which is most likely due to the Al–Cu bi-metal preparation not being conducted in an inert or reducing atmosphere. As shown in Figure 3, pores were also observed near the Al–Cu interface, and these are attributed to the sample cutting and polishing process.

**Figure 4:**
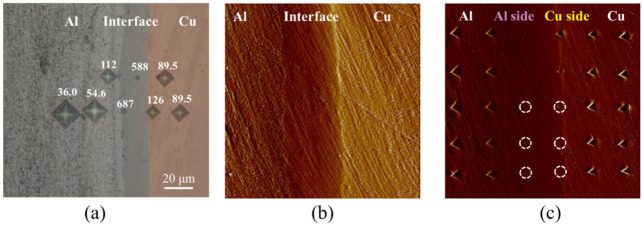
(**a**) The microhardness indents across the interface, (**b**) the interface after polishing, and (**c**) the interface with nanoindentation arrays.

The residual imprints from the microindentation tests across the Al–Cu interface are shown in Figure 4a. For the locations from which the hardness measurements were made, the microhardness values were found to vary. On the Cu side of the interface, the hardness increased from 89.5 Hv_25_ to 126 Hv_25_. In the interface region, the hardness was significantly higher (687 Hv_25_, 588 Hv_25_). On the Al side of the interface, the hardness gradually decreased from 112 Hv_25_ and 54.6 Hv_25_ to 36.0 Hv_25_.

The surface for nanoindentation testing was prepared by using the standard polishing method, and the surface roughness was reduced to about 20 nm, as shown in Figure 4b. The residual imprints from the nanoindentation testing are shown in Figure 4c. Since the indentations were conducted under constant maximum load conditions, the size of indents is observed to be inversely proportional to the nanohardness values of the indented locations. Hence, smaller indents were observed in the interface region while larger indents were observed in the Al and Cu matrix regions. Meanwhile, the edges of indents on the Cu side were much brighter than those on the Al side, indicating that the pile-up effect in Cu was more significant than on the Al side. This phenomenon corresponds to the higher degree of work hardening observed in Cu [29,30].

**Figure 5:**
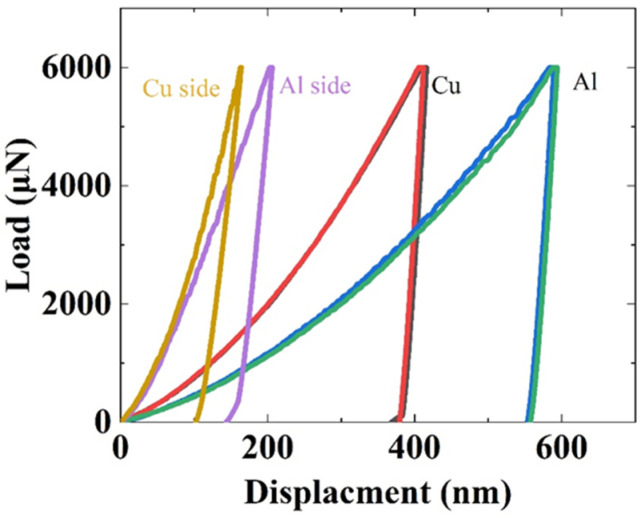
The load-displacement curves of the indents shown in Figure 4c.

The load–displacement curves obtained from the indentation tests are shown in Figure 5. The loading–unloading curves of all tests were smooth without any kink-in or pop-in, ensuring that neither cracks nor phase transformations occurred during the indentation. The indentation load–displacement curves indicate that the most compliant response is obtained in the Al matrix region while a stiffer response is obtained in the Cu matrix region. The indentation curves are significantly stiffer in the interface region, with those regions closer to the Cu side exhibiting relatively higher stiffness.

The nanohardness and the elastic modulus of each indent were calculated based on the Oliver–Pharr method [8,9]. The contacted area (Ac) function, which had been calibrated before the tests, was identified as follows:

(1) $$A_{c}=c_{0}h_{c}{}^{2}+c_{1}h_{c}+c_{2}h_{c}{}^{\frac{1}{2}}+c_{3}h_{c}{}^{\frac{1}{4}}+c_{4}h_{c}{}^{\frac{1}{8}}+c_{5}h_{c}{}^{\frac{1}{16}}$$

where $c_{0}$ = 24.5, $c_{1}$ = −20,092, $c_{2}\;$ = +184,000, $c_{3}$ = −19,931,500, $c_{4}$ = +51,618,900, and $c_{5}$= −33,765,500.

The nanohardness was calculated following Equation (2) [8]:

(2) $$H=\frac{P_{max}}{A_{c}}$$

where $P_{max}$ was 6000 μN. Then, the reduced modulus, *E_r_*, and the elastic moduli, *E*, of the interface region at different locations were calculated using Equations (3) and (4) [8]:

(3) $$E_{r}=\frac{\sqrt{\pi }S}{2\sqrt{A_{c}}}$$

(4) $$\frac{1}{E_{r}}=\frac{1-\nu^{2}}{E_{Al-Cu}}+\frac{1-\nu_{i}{}^{2}}{E_{i}}$$

where S is the unloading slope obtained from the experimental unloading curve, using a standard method, $E_{Al-Cu}$ is the elastic modulus of the indented region, and $E_{i}$ (1140 GPa) and $\nu_{i}$ (0.07) are the elastic modulus and Poisson’s ratio of the diamond Berkovich indenter, respectively. The local elastic modulus, $E_{Al-Cu}$, is determined with Poisson’s ratio ν = 0.3. The interface region exhibits much higher hardness and elastic moduli values than those of the Al or Cu matrix regions: the averaged nanohardness of the interface region near the Al side, and Cu side were observed to be 7.64 GPa and 9 GPa, respectively, while the averaged elastic modulus of the interface near the Cu side and Al side were found to be 172.6 GPa and 135GPa, respectively.

## 4. Modeling and Simulation

### 4.1. Intermetallic Compounds at the Interface

The elastic moduli of the intermetallic compounds AlCu, Al_4_Cu_9_, and Al_2_Cu that are observed to form near the Al–Cu interface were calculated using the first-principle approach. All the calculations were performed with Quantum Espresso code [31] based on the density functional theory (DFT) where the pseudopotentials were of the ultra-soft type. The exchange-correlation functional was described through the generalized gradient approximation with the Perdew–Burke–Ernzerhof parameters [32,33]. The Broyden–Fletcher–Goldfarb–Shanno (BFGS) approach was applied to automatically relax the internal coordinates of atoms to achieve the minimum total energy of the system. In order to ensure the calculation accuracy of different structures, the convergence of the results with respect to cut-off energy and k-points was carefully considered. Based on the convergence test, the cut-off energy value of plane wave functions was set to 40 Ry. The k-points meshes were set to 12 × 12 × 12, 12 × 12 × 13, and 12 × 12 × 12 for AlCu, Al_4_Cu_9_, and Al_2_Cu, respectively, to ensure the balance of accuracy and performance. The convergence tolerance of energy was taken as 0.001 Ry. 

The elastic moduli obtained from the DFT calculations conducted in the present study and the moduli reported from previous studies [34] are listed in Table 2. The elastic moduli obtained from the present study are 181 GPa, 184 GPa, and 145 GPa for Al_4_Cu_9_, AlCu, and Al_2_Cu, respectively. Al_4_Cu_9_ is elastically stiffer than Al_2_Cu, and quite similar to AlCu in that regard. Similar results were also observed in a previous nanoindentation study [35]. While the experimentally observed moduli are different from those predicted by the DFT calculations, the trend in the variation of the elastic moduli across all three intermetallic compounds is captured. There could be several factors that contribute to the differences in the results obtained from DFT calculations and experiments. These include the assumption of perfect crystal structure in the DFT calculations, the effects of grain size of materials, indentation depth, and the geometric shapes of the indenters on the elastic moduli measured in experiments [3,35,36,37].

It is also observed that the elastic moduli of the pure intermetallic phases obtained from DFT calculations or experiments are higher than the moduli obtained from the nanoindentation experiments near the interface regions. This is attributed to the fact that in the nanoindentation experiments, the indented areas may not be 100% pure intermetallics. Given the development of the compositionally graded structure across the Al–Cu interface, it is very likely that a small amount of (softer) matrix material is present in the zone of indentation [20]. A simple rule of the mixture concept can be used to estimate the volume fractions of the softer matrix phase and the harder intermetallic phase [38,39] as follows:

(5) $$E_{Al/Cu\;interface}=V_{int}*E_{int}+V_{P}*E_{P}$$

where $E_{int}$ is the elastic modulus of the intermetallic phase from the DFT calculation, $E_{P}$ is the elastic modulus of Al or Cu, and $E_{Al/Cu\;interface}$ is the elastic modulus of the Al or Cu side of the interface. $V_{int}$ and $V_{P}$ denote the volume fraction of the intermetallic phase and pure metal (Al or Cu), separately. Using a two-phase model where the sum of $V_{int}$ and $V_{P}$ is 100%, the interface near the Cu side is estimated to possess about 12% Cu and 88% Al_4_Cu_9_, while the interface near the Al side is estimated to possess about 87% Al_2_Cu and 13% Al. 

It is noted that the elastic modulus and crystal structure of AlCu are quite close to that of Al_4_Cu_9_. Therefore, in the atomic simulations discussed in the following sections, only the Al_4_Cu_9_ structures were considered.

### 4.2. Molecular Dynamics Simulation

#### 4.2.1. Structure of the Interfaces

**Figure 6:**
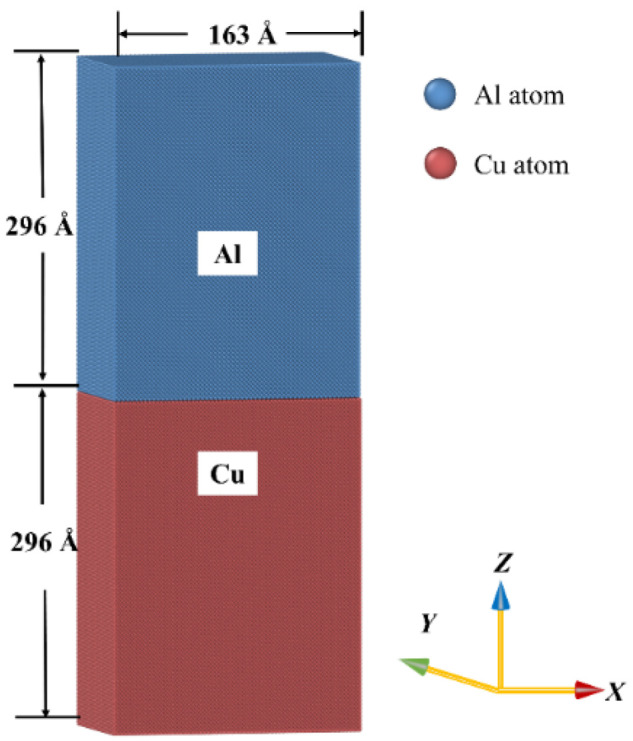
The sketches of the initial atomic model for the Al–Cu interface before diffusion (The contact surface is (001) plane).

MD simulations were conducted using the Large-scale Atomic/Molecular Massively Parallel Simulator (LAMMPS) [40], and the simulated results were visualized using the Open Visualization Tool (OVITO). The simulation system included a copper region (lower part in Figure 6) and an aluminum region (upper part in Figure 6). The unit cell sizes (i.e., Lx × Ly × Lz) of the Cu and Al regions were 163 Å × 36.5 Å× 296 Å and 163 Å × 36.5 Å × 299.7 Å, respectively, along [100], [010], and [001] directions as shown in Figure 6. The embedded atomic method (EAM) potentials from an earlier study [41] were used for Cu–Cu, Al–Al, and Cu–Al atomic interactions to simulate the diffusion process. This EAM has also been used in a previous investigation for Al and Cu alloys [41]. The temperature for the diffusion simulation was held at about 1000 K, which was consistent with the temperature used in the previous experiment [27]. The isothermal–isobaric (NPT) ensemble with a 1 fs time step was used to control the diffusion process. Five layers of atoms at the bottom and the top along Z direction were fixed. Periodic boundary conditions were applied to the unit cell in other directions.

**Figure 7:**
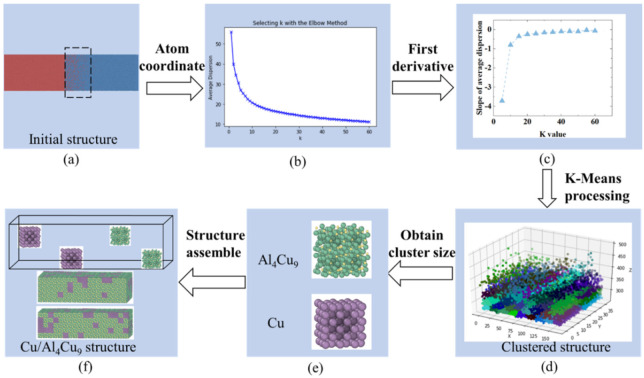
The flow chart of KMeans algorithm to construct the Cu/Al_4_Cu_9_ interfacial structures: (**a**) initial structure, (**b**) K value–average dispersion, (**c**) K value–the slope of average dispersion, (**d**) the cluster sturcuture in Python, (**e**) the cluster size of Cu and Al_4_Cu_9_, and (**f**) constructed Cu–Al_4_Cu_9_ atomic structure.

The atom positions after the diffusion process were predicted in MD simulations. However, the precise formation of intermetallic compounds near the interface regions in the cooling stage could not be directly obtained in the MD simulations. According to experimental observations, the intermetallic dominated zone near the interfaces would typically include a small portion of the matrix metals as well. For example, near the Cu side of the interface, it was observed that more than about 95% Cu was present with less than 5% of Al atoms [42]. As it was difficult to directly determine the cluster size of intermetallics and the cluster distribution of Al in this region, the K-Means clustering algorithm, which is an unsupervised learning model, was invoked, to estimate the cluster size. In the first step, the coordinates of the diffused atoms (such as the coordinates of the Cu atoms between 267–365 Å), in the region highlighted in Figure 7a, were delivered to an in-house developed Python code. The average Euclidean squared distance from the centroid of each cluster, which was named average dispersion, was computed and found to depend on the cluster size. The K number denotes the number of non-overlapping distinct clusters or subgroups within the diffusion zone. The correlation between the average dispersion and the K value is shown in Figure 7b. As the K value increases, the average dispersion reduces to 10. Then, by using the first derivative of the average dispersion–K value correlation, the slope of the average dispersion was obtained, as shown in Figure 7c. The slope of average dispersion is stable when the K number is over 30. Therefore, we chose the K value of 50, and the corresponding clustered structure is shown in Figure 7d, where the atoms belonging to the same group were assigned the same color. Based on the cluster structure, the smallest average group size was estimated to be around 9.35 Å. Therefore, the final cluster size was determined as 9.35 Å, as shown in Figure 7e. The diffusion zone was divided into 990 small regions according to the obtained cluster size. Among the 990 clusters, 871 were assigned with the Al_4_Cu_9_ structures, and the rest were assigned with the pure Cu structures for the interface on the Cu side. In addition, we assumed that pure Al or Cu clusters were homogeneously distributed within this small region. The final interfacial structure is shown in Figure 7f. With a similar approach, 861 groups were assigned with the Al_2_Cu structures for the diffusion zone near the Al side. We denote the region between the Cu matrix and the Al_4_Cu_9_ intermetallic dominated interface as the Cu–Al_4_Cu_9_, and the fine region between the Al matrix and the Al_2_Cu intermetallic dominated interface as Al_2_Cu–Al. In order to obtain robust results, three models were generated for each interface with different (random) distributions.

#### 4.2.2. Effects of Annealing Temperature on the Interfacial Strength

**Figure 8:**
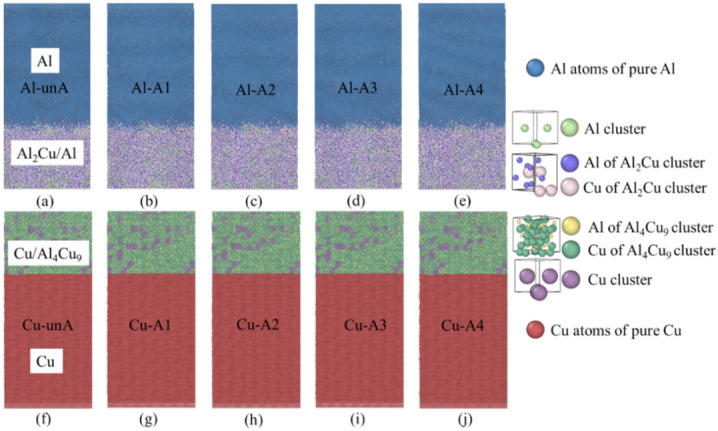
The constructed Al_2_Cu–Al interfacial structures (**a**–**e**) and Cu–Al_4_Cu_9_ interfacial structures (**f**–**j**) before annealing and annealed at different temperatures.

As the as-diffused interface generally possesses high internal stress, an annealing treatment is given to reduce the internal stress [27]. In the present study, an annealing treatment was also conducted on the constructed interface using MD simulation. The atomic structures were cooled and relaxed at 300 K for 100 ps until the pressure was stable. Then, the atomic systems were heated up to 373 K, 473 K, 573 K, and 673 K at 0.1 K/ps and kept for another 50 ps. The atomic structures were slowly cooled down to 300 K in the final step. The heat treatment process was conducted in the NPT ensemble with a 0.1 MPa pressure. After the annealing treatment, it was observed that the interface on the Cu side was almost unchanged, while the diffusion zone was mainly located in the region near the Al side, as shown in Figure 8. The as-diffused structures were named Al-unA/Cu-unA, and the four annealed structures were designated as Al/Cu-A1 (373 K), Al/Cu-A2 (473 K), Al/Cu-A3 (573 K), and Al/Cu-A4 (673 K).

**Figure 9:**
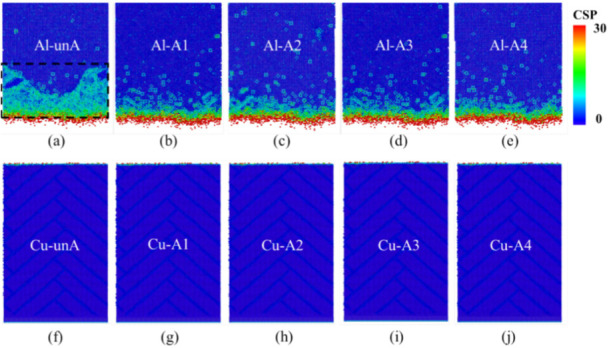
The centrosymmetry parameter (CSP) analysis of Al side of Al_2_Cu–Al structures in (**a**) as-diffused state and (**b**–**e**) annealed states, and Cu side of Cu–Al_4_Cu_9_ structures in (**f**) as-diffused state and (**g**–**j**) annealed states.

Centrosymmetry parameter (CSP) analyses were conducted on the unannealed and the annealed structures. The CSP is used to evaluate the degree of lattice disorder for atomic structures [43,44]. A higher CSP value indicates a more disordered structure. As shown in Figure 9a–e, the interface on the Al side was more disordered with higher CSP values, while the interfaces were relatively smooth and more ordered (with low CSP values) on the Cu side, as shown in Figure 9f–j. More disordered atoms were observed in the unannealed structure, as highlighted in the black box in Figure 9a. Fewer disordered atoms were observed amongst the annealed structures (Figure 9b–e). Thus, due to the microstructural changes that are observed at the interface, it is reasonable to expect the mechanical properties of the interfaces could be influenced by the annealing treatment on the Al side interface.

**Figure 10:**
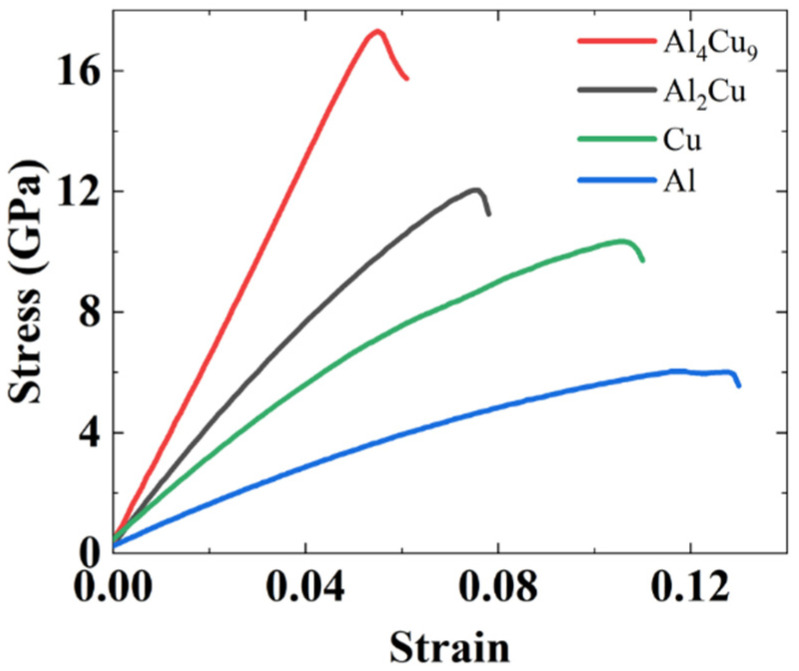
The stress–strain curves and fitted elastic modulus of the pure intermetallic compounds (Al_4_Cu_9_ and Al_2_Cu) and metals (Cu and Al).

In order to assess the effects of annealing on the mechanical properties of the interfaces, tension tests, and shear tests were simulated on the interfacial structures. The conjugate gradient method was used to optimize the annealed structures until the system reached a stable state under the NPT ensemble. Then, tensile and shear deformations were imposed in the NVT ensemble. The strain rate in tensile and shear tests was controlled at 10^9^/s. In a previous study, it was observed that strain rates between 10^9^/s to 10^7^/s did not affect stress–strain behavior [22]. While multiple interatomic potentials for the Al–Cu systems have been reported, we utilized the potential function from Apostol (angular-dependent interatomic potential, ADP) [45] and Liu (EAM potential) [46] for the Al_4_Cu_9_ and Al_2_Cu intermetallics, respectively. For validation purposes, tensile simulations were conducted for pure Cu, pure Al, pure Al_4_Cu_9_, and pure Al_2_Cu, and the engineering stress–strain curves that were obtained are presented in Figure 10. The elastic moduli (*E*) of these four materials followed the same trend as observed in the experimental results: *E*_Al4Cu9_ (317 GPa) > *E*_Al2Cu_ (185 GPa) > *E*_Cu_ (129 GPa) > *E*_Al_ (66 GPa). Note that the elastic modulus of Al_4_Cu_9_ in the molecular dynamics simulations, which is based on the ADP [45], is the closest value to the one obtained from the DFT calculation. 

##### The Tensile Strength

**Figure 11:**
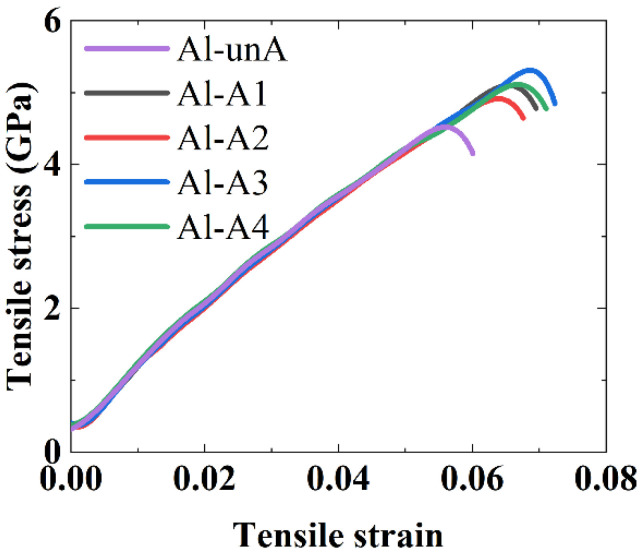
The tensile stress–strain curves of the Al_2_Cu–Al structures before annealing and at different temperatures of annealing.

The tensile engineering stress–strain curves of the Al_2_Cu–Al interfacial structure obtained from the MD simulations are shown in Figure 11.

The loading curves in the tensile tests were relatively smooth because EAM potential functions were adopted for both Al and Al_2_Cu. It is observed that the annealing treatment has a significant effect on the interfacial tensile strength of the structures near the Al side. The interfacial tensile strength increased from 4.56 GPa in the unannealed Al interface structure (Al-unA) to 5.2 GPa in the Al interface that was annealed at 373 K (Al-A1). The highest tensile strength of 5.3 GPa was found in Al-A3 structure which was annealed at 573 K. As the current atomic simulations are focused on a small volume of material (with much fewer defects), the stresses generated in the simulations are much higher than the ones observed in experiments where the material has typically more defects.

Meanwhile, the failure strain increased from 5.7% to 7.0% as the annealing temperature was increased from 373 K to 573 K. In comparison to the Al interface structure that was annealed at 573K, the ultimate tensile stress and the failure strain were slightly reduced for the Al-A4 structure (which was annealed at 673 K). Annealing effects at temperatures higher than 673 K were not considered in this study, as the intermetallic compounds are not thermodynamically stable and are expected to dissolve at those temperatures, according to the Al–Cu phase diagram and experimental observations [26].

**Figure 12:**
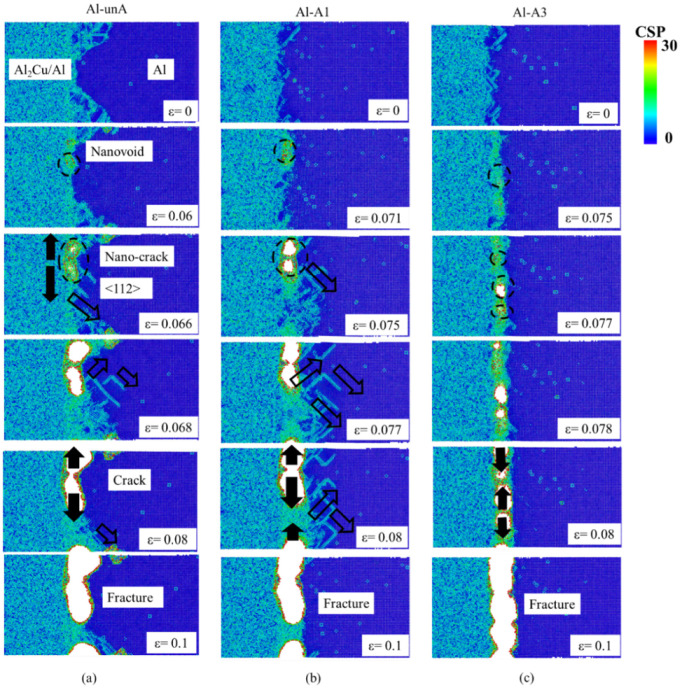
CSP analyses for (**a**) Al-unA, (**b**) Al-A1, and (**c**) Al-A3 at different strain values. (The unfilled arrows denote the dislocation propagation direction, and filled arrows denote the crack propagation direction.)

The evolution of the fracture process at the Al interface was further investigated using the CSP analyses, and the results obtained in three structures, Al-unA, Al-A1, and Al-A3, are shown in Figure 12.

In the tensile simulations of the unannealed interface, because interfacial stresses are generated near the interface in the cooling process, stacking faults and atoms with relatively high CSP values were found near the interface. During the tensile straining process, the CSP values of atoms near the interface were further increased. Compared to the initial un-stressed atomic structure, a thicker layer of atoms with high CSP layer values was formed. Then, nanovoids were generated near the interface when the strain reached about 6%. The nanovoids were generated in the locations bordering near perfect structures (i.e., with a low CSP value) and the region with a high CSP value (or a high stacking fault density) in all three atomic models. It is hypothesized that stress release at the highly strained interface between the perfect regions and those with atoms with high CSP values is the driving force that contributes to the formation of nanovoids in those locations. 

With increasing levels of stress, the size of the nanovoids increased and plastic deformation was also observed to occur. New dislocations were formed near the interface and propagated along the <112> direction in the pure Al structure when the strain reached 6.6% (Figure 12a). As the tensile deformation increased, the nanovoids linked up and some dislocations were also observed to move and converge with the nanovoids. The merging of the nanovoids led to the formation of incipient cracks. When the tensile strain reached 6.8%, the cracks quickly expanded along the interface, and the overall stress was reduced. With increasing strain, the cracks were observed to increase in size and total fracture occurred at a strain of about 10%. 

In the case of annealed interfaces (Figure 12b,c), nanovoid formation, dislocation generation, and crack formation processes were also observed. However, nanovoid formation occurred at tensile strains which were higher than those observed in the unannealed structures. Relative to the unannealed structure, more dislocation activity was also observed in Al-A1. This was attributed to a higher population of dislocations that was generated at the end of the annealing process prior to the tensile simulations. In contrast, few new dislocations were generated in Al-A3. 

Overall, the fracture process of the interface can be summarized as following five steps: nanovoids initiation, new dislocations generation, void coalescence, crack propagation, and total fracture. In general, the annealing treatment was observed to reduce the degree of disorder in the atoms near the interface and increase the strain that corresponded to the onset of nanovoid generation, which resulted in improved strength of the interface. The fracture surface was observed to be relatively smooth, as noted in a prior experimental study [21].

**Figure 13:**
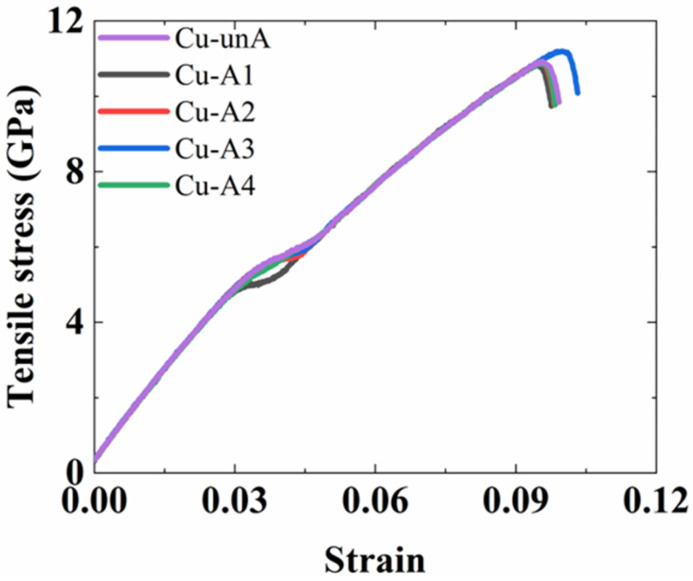
The tensile stress–strain curves of the Cu–Al_4_Cu_9_ structures before annealing and at different temperatures of annealing.

However, in the tensile simulations of the Cu interface (Figure 13), very little effect of annealing was observed. Only a slight increase in the tensile strength was observed in the case of Al-A3. The kink-in observed at about 4% strain in the loading curve was attributed to the use of the angular dependent atomic potential (ADP) for the Al_4_Cu_9_ phase.

**Figure 14:**
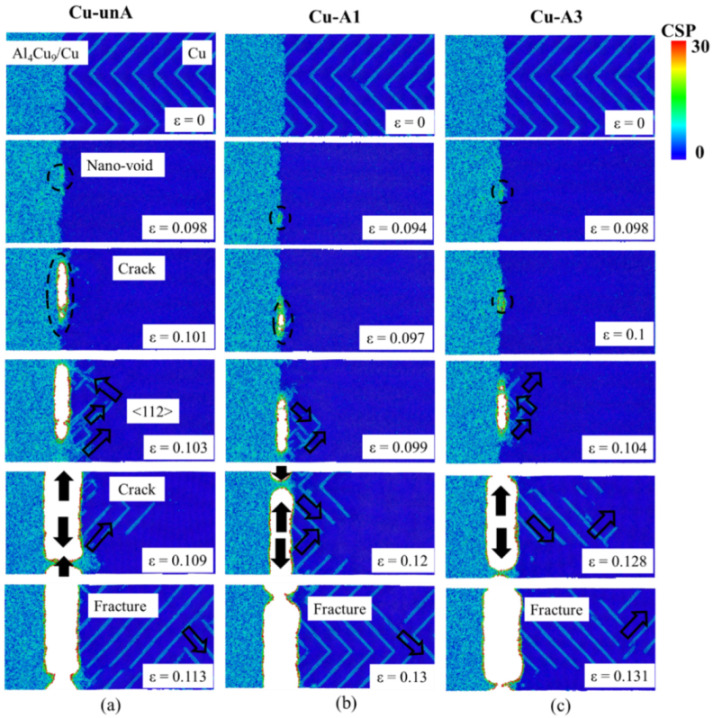
CSP analyses for (**a**) Cu-unA, (**b**) Cu-A1, and (**c**) Cu-A3 at different strain values. (The unfilled and filled arrows denote the dislocation and crack propagation direction, respectively).

Upon examining the atomistic processes that contribute to the development of fracture at the Cu interface (Figure 14), it is evident that the nature of the interface which was smooth in the unannealed remains unchanged in the annealed structures as well. As the annealing treatment did not significantly affect the atomic structure of the interface on the Cu Side, the fracture processes observed in the unannealed and the annealed structures were quite similar. 

While the atomistic processes that preceded the failure of the interface were similar to those observed in the Al interface, the formation of cracks due to the coalescence of nanovoids was observed to occur at higher tensile strains (and proportionately higher tensile stress) in the Cu interface (Figure 14).

**Figure 15:**
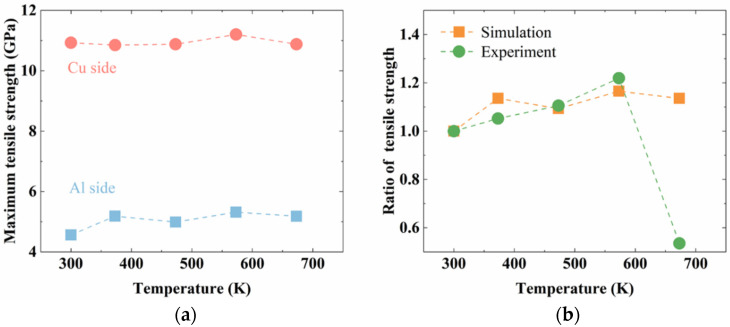
(**a**) The tensile strength of Cu–Al_4_Cu_9_ structures, and Al_2_Cu–Al structures, and (**b**) the comparison between the simulated tensile strength ratio (strength of annealed structure over the strength of unannealed structure) and the experimental one.

Figure 15a summarizes the tensile strength observed in all the interface structures. The strength of the Al interface was consistently lower than that of the Cu interface for both the unannealed and the annealed structures. As shown in Figure 12 and Figure 14, failure of the Al–Cu interface is first initiated at a lower stress at the Al interface. Thus, the strength on the Al side determines the strength of the entire Al–Cu interface.

By comparing the ratio of the tensile strength of the annealed structure and the tensile strength of the unannealed structure obtained in this study with the experimental results reported in a previous study [28] (Figure 15b), it is evident that similar trends are observed. The strength increased with a higher annealing temperature and reached the highest value at 573 K. The ratios from experiments are quite close to the values obtained from simulations. The larger discrepancy that was observed between the results obtained from simulations and experiments at 673 K was attributed to the dissolution of the intermetallic phases in the experiments, which were not captured in the simulations. In summary, the simulations of the present study successfully demonstrate the effects of annealing temperature on the tensile strength of the Al–Cu bi-metallic interfaces.

##### The Shear Strength

**Figure 16:**
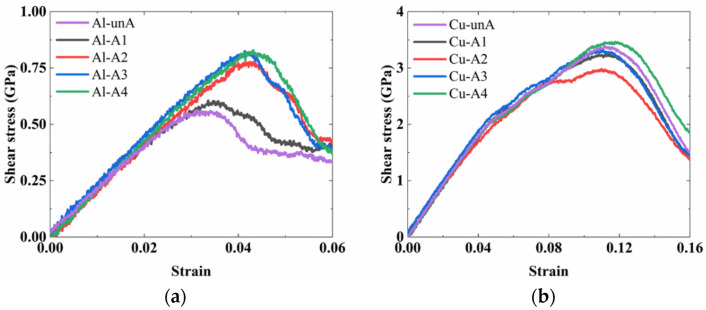
The shear stress–strain curves of the interfacial structures before annealing and at different temperatures of annealing of (**a**) Al_2_Cu–Al structures and (**b**) the Cu–Al_4_Cu_9_ structures.

The shear stress–strain curves of the Al_2_Cu–Al interface (i.e., the Al interface) and the Cu–Al_4_Cu_9_ interface (i.e., the Cu interface) obtained from the MD simulations are shown in Figure 16.

As discussed in the previous section, the annealing treatment releases internal stress at the interface and also reduces the amount of disorder, esp., at the Al_2_Cu–Al interface. Similar to the effect that was observed in the case of tensile strength, the shear strength of the interface was also influenced by the annealing temperature. The shear strength was increased by about 46% when comparing the lowest shear strength (0.56 GPa) obtained in the as-diffused/unannealed structure with the highest shear strength (0.82 GPa) obtained in the structure that was annealed at 573 K.

The stress–strain curves capturing the shear deformation of the Cu–Al_4_Cu_9_ structure are shown in Figure 16b. Unlike in the case of the Al interface, the shear strength of the Cu interface decreased initially as the annealing temperature was increased to 373 K and 473 K, and then increased as the annealing temperature was increased further to 573 K and 673 K. The highest shear strength was observed for the Cu interface that was annealed at 673 K. The shear fracture strain was more than 10%, which was much higher than the tensile fracture strain. Since it was difficult to track the crack initiation in the shear stimulation, CSP analyses of shear fracture were not conducted.

**Figure 17:**
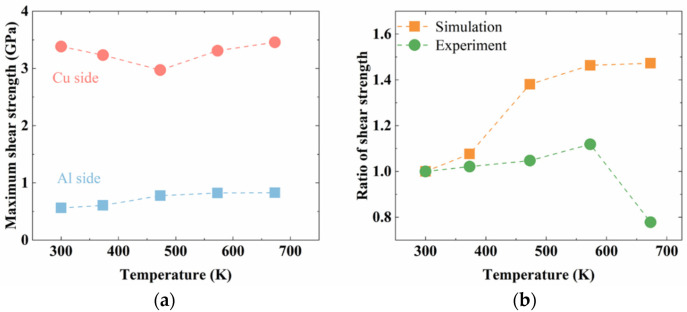
(**a**) The shear strength of Cu–Al_4_Cu_9_ structures, and Al_2_Cu–Al structures, and (**b**) the comparison between simulated shear strength ratio (strength of annealed sample over the strength of unannealed sample) and the experimental ones.

Overall, the shear strength of the Al interface was consistently lower than the strength of Cu interface in the unannealed and annealed conditions as summarized in Figure 17a. The shear strength is closely correlated to the Shockley partial dislocation. Because the Shockley partial dislocation possesses the same Burgers vector direction as that of the crystal slip, a greater dislocation movement and an earlier yield phenomenon should be expected in the shear simulation if the structure has a higher dislocation density. After the materials processing, the Cu-A2 structure has the most Shockley partial dislocations, and the lowest shear strength was seen in Figure 17a.

By comparing the ratio of the shear strength of the annealed structure and the shear strength of the unannealed structure obtained in this study with the experimental results reported in a previous study [28] (Figure 17b), it is evident that similar trends are observed. As the MD simulations do not capture the dissolution of the intermetallic phases at higher annealing temperatures, the model predictions for the shear strengths at higher temperatures are higher than those that are observed in experiments. Thus, the simulations in the present study successfully capture the trends observed in the effects of annealing temperature on the shear strength of the Al–Cu bi-metallic interfaces as well.

### 4.3. Comparison with Experiments

The MD simulations in the present study have successfully predicted that annealing temperature around 573 K is optimal for enhancing the strength of the Al–Cu bi-metallic interface. Furthermore, the MD simulations have provided clear insights into the relative strengths of the interfaces on the Al side and the Cu side, with Al side interface being identified as the weaker interface. Thus, enhancing the strength of the Al-side interface is expected to enhance the strength of the overall Al–Cu bimetallic interface. 

Nano and microindentation experiments across the interface regions have clearly provided experimental evidence that the Al-side of the interface is weaker than the Cu-side as it is more compliant and exhibits lower hardness compared to the Cu-side of the interface, which corroborates with the insights obtained from the MD simulations. 

It is acknowledged that the actual shear and tensile strengths measured in experiments could exhibit some variability depending on the differences in experimental test conditions and in sample preparation steps. Liu et al. [20] have reported that the tensile strength/shear strength ratio was about 2, and our earlier experimental study [28] reported that the tensile strength/shear strength ratio was over 10. The MD simulations in the present study indicate that the tensile strength/shear strength ratios on the Al and Cu sides are slightly different, and the average ratio is between 5 and 6. This discrepancy between the results of the MD simulations and prior experimental results could be due to the small sample size that can be modeled using MD simulations which do not allow for some of the microstructural features to be captured in the simulations, such as micronscale and mesoscale defects that could be present at the interfaces and other microstructural aspects, such as grain misorientation effects. Despite the limitations of the MD simulations, the present study has demonstrated that the annealing process enhances the quality of the interface by reducing interfacial defects and thus enhances the strength of the bi-metallic interface. 

Overall, there is an opportunity for further developing the MD simulation framework presented in this study to couple the effects of annealing temperature and pressures to model possible phase transformation effects, if any, and optimize the casting and annealing process to achieve the highest possible interfacial strength.

## 5. Conclusions

Within the context of designing and developing lightweight energy-efficient systems in the transportation and electrical power sectors, there is a need for replacing heavier metallic components with lighter components (such as aluminum). The process of lightweighting structures often requires joining or welding such lightweight components in the structural systems. In order to obtain long term durability of the welded or joined structures, the interfaces in such welded or joined structures need to be as strong as possible. For optimizing the manufacturing conditions that would enhance the strength of such bi-metallic interfaces, there is a need to obtain a fundamental understanding of the evolution of the microstructures, particularly diffusion processes and the defect evolution processes which occur at the nanoscale at these interfaces, and their influence on the strength of the interfaces.

Hence, the present study was focused on developing a molecular dynamic simulations-based modeling framework for understanding atomic configuration evolution in the deformation of the Al–Cu bi-metallic interfaces. The principal conclusions of the present study are summarized as follows:

1. The Al–Cu as-cast and rolled Al–Cu interface exhibits a compositionally graded structure with two distinct interfaces—one on the Al-side which is rich with the intermetallic Al_2_Cu and another one on the Cu-side which is rich with the intermetallic Al_4_Cu_9_.
2. The mechanical failure of the Al–Cu interface takes place at the Al-side of the interface which is weaker than the Cu-side of the interface.
3. Micro- and nanoindentation experiments on the graded Al–Cu interfaces also confirm that the Al-side of the Al–Cu interface is mechanically weaker than the Cu-side.
4. Centrosymmetry parameter analyses and dislocation analyses that are used to understand the microstructural features that influence deformation behavior leading to the failure of the Al–Cu interfaces reveal that increasing the annealing temperature increases the quality of the interface by reducing the stacking fault density at the Al-side of the interface, suppresses the generation of nanovoids, which are precursors for the initiation of fracture at the Al-side of the interface, and thus increases the tensile and shear strengths of the interfaces.
5. The trends predicted by the MD simulations for the increase in the tensile and shear strengths of the interfaces observed with annealing treatments are in agreement with experimental results.

In future studies, the strength of the intermetallic layer will be predicted using micro and mesoscale methods, such as the crystal plasticity finite element method. The voids and their distribution, and grain misorientation effects, will be included.

## Figures and Tables

**Table 1:** The element composition in weight percentage (wt. %) and atom percentage (at. %) of the locations highlighted in Figure 3.

Element	wt.% near Cu	at.% near Cu	wt.% near Al	at.% near Al
C	1.11	4.50	1.18	3.69
O	0.80	2.43	0.91	2.14
Al	17.37	31.44	45.53	63.27
Cu	balance	balance	balance	balance

**Table 2:** The elastic modulus (E) of the three phases: DFT calculation and experiment (exp).

Phase	*E* (GPa)	*E* (GPa) [35]	*E* (GPa) [3]	*E* (GPa) [36]	*E* (GPa) [37]
AlCu	184.0 (DFT)		227.9 (exp)		
Al_4_Cu_9_	181.3 (DFT)		254.7 (exp)		
Al_2_Cu	145.2 (DFT)	112.0 (exp)	112.4 (exp)	132.5 (DFT)	169.6 (DFT)

## Data Availability

Not applicable.
